# AMN Congress 2022 – Report of the panel on the future focus of the Academy for Multidisciplinary Neurotraumatology

**DOI:** 10.25122/jml-2022-1013

**Published:** 2022-07

**Authors:** Alexandra-Mihaela Gherman, Andreea Strilciuc, Dafin Fior Muresanu

**Affiliations:** 1. RoNeuro Institute for Neurological Research and Diagnostic, Cluj-Napoca, Romania; 2. Department of Neuroscience, Iuliu Hatieganu University of Medicine and Pharmacy, Cluj-Napoca, Romania

"The AMN is an ambitious medical society – what should be its focus?" **–** is the challenge question of the third Panel taking place on Day 2 of the 19^th^ AMN Congress, to which the following panelists provided extremely daring answers:

- Volker Hömberg (Germany);
- Jongmin Lee (South Korea);
- Lynne Lucenna (The Philippines);
- Nicole von Steinbüchel (Germany);
- Cristian Matula (Germany);
- Dafin Muresanu (Romania);
- Pieter Vos (The Netherlands);
- Johannes Vester (Germany).

The setting of the discussion was dynamically constructed by Prof. Dafin Muresanu around the vision and mission of the AMN, *i.e*., the advancement of neurotraumatology in research [1], clinical activity and education, a multidirectional purpose intended to be met and further built upon by focusing on a four-pillar structure and what particular developments all these already encapsulate from what it is so far a twenty years' itinerary:

- **Science** – a pillar full of scientific heritage and scientific future, *e.g*., the CAPTAIN studies and its derivative non-interventional studies whose very appealing methodology will bring enough evidence to push the field of neurotrauma, and C-RETURN which will come to confirm, using a cohort of more than 480 patients, all the findings generated by the CAPTAIN project;
- **Education** – with consistency and complexity given by the annualy organized AMN Congress, but also by the international webinar series and various workshops developed (*e.g*., the NTSC-Vienna event in the near future with its unique simulation feature offers an approach that needs to be tackled further); in addition, the international webinars will be granulated in a consortium of societies so that an emphasis will be placed on the AMN collaboration with WFNR (World Federation of Neurorehabilitation) and EFNR (European Federation of Neurorehabilitation);
- **Clinical practice** – a pillar in motion, focusing now on PRESENT (Patient REgistry – Short Essential NeuroTrauma), a project that will bring more clarity into the flow of TBI patients and the data to be collected, but also on developing other studies in order to move forward in the TBI field, like delivering key guidelines similar to those delivered by the European Academy of Neurology (EAN) on stroke-related issues, early rehabilitation, and pharmacological support in acute stroke patients. As such, a new TBI guideline project is envisaged, based on the Grading of Recommendations, Assessment, Development and Evaluation (GRADE) system (generated by using the PICOST (Population, Intervention, Control, Outcomes, Study design and Timeframe) approach, *i.e*., study of population and care setting, types of interventions and comparators, *e.g*., standard of care and treatment, together with a stratified approach by intensity, with a focus on outcomes related to efficacy and safety measured at different timelines, *i.e*., 30, 90, 180 and 360 days) as an instrument to clarify evidence and the strength of recommendations used in the clinical management of moderate-severe TBI.
- **Advocacy** – an instrument used to enhance the connectivity of AMN at the European and international level (*e.g*., the MoUs with EAN and WFNR, and alongside the latter, the special focus on the collaboration with the World Health Organization (WHO)) in such a way as the Academy to become a lead voice amongst the neurotraumatology societies.

As another background setting has been already put in place for the Academy, *i.e*., a refurbished AMN website with upgraded content and a rich and attractive blog section, among others, Prof. Dafin Muresanu turns to the importance of the advocacy of making AMN's voice heard because “If there is no voice, there is no change” and actions are not visible. For these things to happen, there is a need to also look at other societies that are promoting and looking towards shaping themselves in the future while maintaining the focus of the AMN, namely its concern for TBI and keeping alive the field of general neurology, alongside other renowned European academies. In other words, to avoid losing the integration and integrative approach against all the subspecialties academic societies, AMN needs to do the other way around and look into the multidisciplinary dimension and integrate everything that is generated from the “extremely fertile soil of neurotrauma”. In addition, based on the own model created and the manpower comprised, AMN needs to become more competitive and use the intellectual development in order to eventually create a very robust stream to change some rigidities in the standardized manner that things are done nowadays.

Nicole von Steinbüchel underlines that the AMN flourished due to the multidisciplinary cooperation which enhances communication (*e.g*., due to CENTER-TBI [2], a multidimensional assessment of TBI was developed, generating instruments that now can be used in all CAPTAIN studies and other trials, enabling knowledge about what is sensitive or not that can be further applied in developing digital applications), actions which further developed its traits of intellectual interchange, discourses, and friendship. All of these become requisite for overcoming all borders, but also motivate young people to join AMN. Prof. Dafin Muresanu underlines here the presence of Lynne Lucenna from the Philippines and Jongmin Lee from South Korea as clear examples of AMN's geographical coverage, alongside the historical and intellectual ones. These facts also characterize AMN as a high-level society.

Johannes Vester, president of AMN, uses three instrument questions to point to the future focus of the Academy, *i.e*., What is the hard piece of the society? What is the genetic code? and What is the essence? with the main answer being the multidisciplinary approach. In extenso, it is further needed to bring all the branches together to create a higher level, a meta intelligence level in research and practical work to the benefit of patient's future. Equally, it is important to build a network of exchanges that would only be possible if there is trust and confidence, if communication lines are open, ideas are shared with no fear, perspectives are shared and brought together, and finally, all of these could lead to changing mindsets.

In his turn, Cristian Matula points to the fact that the beauty of the multidisciplinary team is what made AMN so strong, yet multidisciplinarity is something that one must live, and for that reason, specialists like neuro-anaesthesiologists, neuroradiologists, traumatologists [3] should also be involved in AMN activities as what counts, at the end of the day, is the result for the patient, the benefit for him. For this purpose, AMN needs to build on visionary people, on its heterogeneity which, if stronger, could sharpen up the Academy and enable it to take important and interesting steps for the future. In connection to this, Pieter Vos highlights the importance of the annual congress as means of focusing on developments in the field of brain injury but should AMN intend to step up to a larger scale, it should also consider the release of a “product” in order to receive funding.

Connected to all of the above, Jongmin Lee and Lynne Lucenna speak about their efforts to develop AMN mirror-like societies in their own countries, the first one having as reasoning the small number of specialised academic societies in South Korea, and the latter to put into practice her theory regarding the principles of task-sharing and task-shifting on neurotrauma [4, 5] that is meant to provide solutions to the scarcity of neurotrauma within the hospital she functions. Moreover, with respect to PRESENT, Lynne Lucenna expresses her wish to have AMN as a partner in developing a similar registry for the Brain and Spine Centre in the process of being set up by her, a facility that will also have a Head Trauma Unit (operating room, recovery room, *etc*.). Pointing out that, in general, psychologists are not taken into consideration when it comes to neurorehabilitation, Nicole von Steinbüchel offers Lynne Lucenna the necessary support to develop something together in this field in the near future.

Coming back to his statements, Cristian Matula indicates that a unique ‘selling point' for AMN would be to bring together the best of what the people really need, *i.e*., in order to make a difference, AMN should offer a wide variety of products placed onto a platform-translating into support, ideas, *etc*. to really respond to the needs of people. In addition, C. Matula believes that it is extremely important for the AMN to add another pillar to its structure, *i.e*., teaching people, a component that can bring in and train younger people as new generations need to be guided.

Volker Hömberg adheres to the statements of the previous panelist by adding that also WFNR is looking for younger people, especially when it comes to developing easily accessible digital applications, *i.e*., low-key registries for patients and data generation.

Finally, Prof. Hömberg draws the attention again to the open-mindedness of the World Health Organization concerning neuro-problems, pointing to the fact that the structural intervention and interaction of WFNR and AMN with WHO will focus on the domains of neurology, brain health new initiatives, and also epilepsy and other neurological problems. Of course, all these will also support the portfolio increase of the Academy for Multidisciplinary Neurotraumatology, translating into products derived from congresses [6, 7], multiple series of webinars, and a variety of courses, either digital or live.

## References

[1] Khellaf A, Khan DZ, Helmy A (2019). Recent advances in traumatic brain injury. J Neurol.

[2] Centre-TBI Traumatic Brain Injury Fact sheets and Policy Brief.

[3] Sundstrøm T, Grände PO, Luoto T, Rosenlund C (2020). Management of severe traumatic brain injury: evidence, tricks, and pitfalls. Springer Nature.

[4] Robertson FC, Esene IN, Kolias AG, Khan T (2019). Global Perspectives on Task Shifting and Task Sharing in Neurosurgery. World Neurosurg X.

[5] Robertson FC, Esene IN, Kolias AG, Kamalo P (2019). Task-Shifting and Task-Sharing in Neurosurgery: An International Survey of Current Practices in Low-and Middle-Income Countries. World Neurosurg X.

[6] Cretoiu D, Livint Popa L (2019). 17th Congress of the Academy For Multidisciplinary Neurotraumatology: 11-12 April 2019 | Platinia Conference Hall | Cluj-Napoca | Romania. Journal of medicine and life.

[7] Cretoiu D (2020). 18th Congress of the Academy for Multidisciplinary Neurotraumatology: 26-28, February 2020 | Cairo | Egypt. Journal of medicine and life.

